# Paxilline Prevents the Onset of Myotonic Stiffness in Pharmacologically Induced Myotonia: A Preclinical Investigation

**DOI:** 10.3389/fphys.2020.533946

**Published:** 2020-11-23

**Authors:** Kerstin Hoppe, Tina Sartorius, Sunisa Chaiklieng, Georg Wietzorrek, Peter Ruth, Karin Jurkat-Rott, Scott Wearing, Frank Lehmann-Horn, Werner Klingler

**Affiliations:** ^1^ Department of Anesthesiology, Intensive Care Medicine and Pain Therapy, Goethe University, Frankfurt University Hospital, Frankfurt, Germany; ^2^ Department of Anesthesiology, Intensive Care Medicine and Pain Therapy, Wuerzburg University Hospital, Wuerzburg, Germany; ^3^ Department of Pharmacology, Toxicology and Clinical Pharmacy, Institute of Pharmacy, University of Tuebingen, Tuebingen, Germany; ^4^ Division of Neurophysiology in the Center of Rare Diseases, Ulm University, Ulm, Germany; ^5^ Faculty of Public Health, Khon Kaen University, Muang Khon Kaen, Thailand; ^6^ Institute for Molecular and Cellular Pharmacology, Innsbruck Medical University, Innsbruck, Austria; ^7^ Division of Experimental Anesthesiology, University Medical Center Ulm, Ulm, Germany; ^8^ Institute of Health and Biomedical Innovation, Queensland University of Technology, Brisbane, QLD, Australia; ^9^ Department of Conservative and Rehabilitation Orthopedics, Faculty of Sport and Health Science, Technical University of Munich, Munich, Germany; ^10^ Department of Anesthesiology, Intensive Care Medicine and Pain Therapy, SRH Clinincs, Sigmaringen, Germany

**Keywords:** paxilline, NS1608, repetitive firing, myotonia congenita, muscle disease, BK channel

## Abstract

Reduced Cl^−^ conductance causes inhibited muscle relaxation after forceful voluntary contraction due to muscle membrane hyperexcitability. This represents the pathomechanism of myotonia congenita. Due to the prevailing data suggesting that an increased potassium level is a main contributor, we studied the effect of a modulator of a big conductance Ca^2+^- and voltage-activated K^+^ channels (BK) modulator on contraction and relaxation of slow- and high-twitch muscle specimen before and after the pharmacological induction of myotonia. Human and murine muscle specimens (wild-type and BK^−/−^) were exposed to anthracene-9-carboxylic acid (9-AC) to inhibit CLC-1 chloride channels and to induce myotonia *in-vitro*. Functional effects of BK-channel activation and blockade were investigated by exposing slow-twitch (soleus) and fast-twitch (extensor digitorum longus) murine muscle specimens or human musculus vastus lateralis to an activator (NS1608) and a blocker (Paxilline), respectively. Muscle-twitch force and relaxation times (T_90/10_) were monitored. Compared to wild type, fast-twitch muscle specimen of BK^−/−^ mice resulted in a significantly decreased T_90/10_ in presence of 9-AC. Paxilline significantly shortened T_90/10_ of murine slow- and fast-twitch muscles as well as human vastus lateralis muscle. Moreover, twitch force was significantly reduced after application of Paxilline in myotonic muscle. NS1608 had opposite effects to Paxilline and aggravated the onset of myotonic activity by prolongation of T_90/10_. The currently used standard therapy for myotonia is, in some individuals, not very effective. This *in vitro* study demonstrated that a BK channel blocker lowers myotonic stiffness and thus highlights its potential therapeutic option in myotonia congenital (MC).

## Introduction

Myotonia congenita (MC) is an autosomal, hereditary non-dystrophic myotonic disease, which is caused by loss-of-function mutations in the skeletal muscle chloride channel type 1 (CLCN1; Lehmann-Horn et al., 2004). Due to its high conductance, chloride stabilizes the resting membrane potential near the chloride equilibrium potential (Adrian and Bryant, 1974; Steinmeyer et al., 1991). A decrease in chloride conductance, therefore, impairs electrical stability of the myofiber resulting in membrane hyper-excitability and spontaneously occurring action potentials (APs). Additional clinical symptoms of MC include a transient inability of striated muscle fibers to relax normally, causing a slowed relaxation (myotonic stiffness). Myotonic stiffness can be combined with fatigue or even an inability of the muscle to generate normal strength (myotonic weakness). However, exercise decreases muscle stiffness, a phenomenon called “warm-up.”

Mexiletine is the current “standard” therapy for MC, which exerts its therapeutic action through a use-dependent block of voltage-gated sodium channels (Novak et al., 2015). Nevertheless, in some individuals mexiletine is less effective and compliance is insufficient due to adverse events. Hence, investigation of further treatment options is required.

Recently, a selective blockade of slow sodium channels (NaPIC) was reported to improve clinical symptoms of myotonia (Novak et al., 2015). In myotonia, repetitive firing of myofibers causes an accumulation of K^+^ in the transverse tubular system, which depolarizes the membrane sufficiently to initiate self-sustaining AP that results in prolonged (myotonic) contraction (Adrian and Bryant, 1974; Clausen, 2013). This depolarization might trigger an activation of slow persistent inward current (NaPIC), which results in repetitive firing (Tricarico et al., 2005; Dupont et al., 2019). Another recently published treatment option includes retigabine, a KCNQ 5 channel activator, which appears to improve myotonia *via* activation of K^+^ current during action potential trains (Dupont et al., 2019). However, to date, these beneficial effects have only been detected under *in vitro* conditions. This might either be explained by administration of an insufficient dose or due to differential sensitivity to retigabine’s effects on different muscle groups (Dupont et al., 2019). However, retigabine is designed to treat epilepsy and therefore shows high penetrance into the central nervous system and depressant effects. Finally, retigabine causes significant muscle weakness that is associated with worse motor performance *in vivo*.

Another treatment option may be ascribed to target the big conductance calcium (Ca^2+^) and voltage activated potassium channels (BK; KCa1.1 encoded by the KCNMA1 gene). This channel enhances the repolarization by restoring the K^+^-gradient during an action potential due to a behavior similar to that of voltage-gated potassium channels (Kristensen et al., 2006). BK channels are activated when skeletal muscle exhibits hypertonicity, mainly by large membrane depolarization and [Ca^2+^]_i_ increases. They are also considered as a protective factor against oxidative stress, dysfunctional Ca^2+^ homeostasis and abnormal fiber excitability as occurs during ischemia-reperfusion, aging and in neuromuscular disorders (Tricarico et al., 2005). As BK channels are predominantly localized to the transverse (T-tubular) system (Siemer et al., 2000; Jung Nielsen et al., 2003) clinically beneficial effects on individuals suffering from myotonia might be expected from modulating their activity.

Previous studies have suggested that increased potassium levels may, in part counter myotonic stiffness and result in an earlier onset of the “warm-up” phenomenon (Hoppe et al., 2013). Thus, the aim of this study was to investigate whether BK channels might influence clinical signs of myotonia.

The T-tubular membrane of skeletal muscle shows a higher expression of the BK channel (Tricarico et al., 2005; Kristensen et al., 2006). However, expression and characteristics of BK channels differ depending on muscle phenotype. For instance, an elevated expression and activity of BK channels could be identified in slow-twitch muscles fibers in rats, which are characterized by a low Ca^2+^ sensitivity and a lack of response to the BK channel opener acetazolamide, a carbonic anhydrase inhibitor (Tricarico et al., 2005). In contrast, rat fast-twitch muscle fibers showed a low expression and activity of BK channels, with a high sensitivity to Ca^2+^ ions and activation response to BK channel influencing drugs (Tricarico et al., 2005). Therefore, we used a murine model to study the effects of BK channel openers and blockers on slow-witch and high fast-twitch muscle fibers after pharmacologically induced myotonia.

## Materials and Methods

### Mice

Wild type and BK channel-deficient mice (BK^−/−^) were used as an animal model to pharmacologically induce low chloride (gCl^−^) conductance myotonia. Animals were kept in a pathogen-free animal facility area of the university. All experiments were performed *in vitro* after euthanasia by cervical dislocation following CO_2_ narcosis, with a concentration of 35% within a transparent cage, for at least 2 min (Moody et al., 2014; Boivin et al., 2016). All animal experiments were performed according to the guidelines laid by the Welfare Committee University of Ulm and Tubingen as well as the National Institutes of Health. All experiments conform to the principles and regulations described by Grundy (2015).

Mice lacking functional BK channels were bred as previously described (Sausbier et al., 2004; Muheim et al., 2019). In brief, BK channel function was abolished by deleting the pore exon of the KCNMA1 gene, which encodes the α-subunit of the pore forming channel protein (Sausbier et al., 2004). Heterozygous C57BL6 mice were paired with heterozygous SV129 mice yielding the F2 generation with SV129/C57BL6 hybrid background (Sausbier et al., 2004). Litter-matched WT and BK^−/−^ mice of male gender at the age of 10–17 weeks were used for this study.

### Human

Open biopsies of vastus lateralis muscle were taken from eight human donors with suspected malignant hypothermia. All biopsies were treated as “non-malignant hyperthermia susceptible” samples. Donors were referred to our neuromuscular center for muscle biopsy, and specimens were used with written informed consent according the local Ethics Committee. The muscle bundles were dissected into 6–10 strips (length 15–25 mm; width 2–3 mm; weight 120–250 mg).

### Induction of Pharmacological Induced Myotonia

Induction of myotonia was achieved by inhibition of chloride channels by anthracene-9-carboxylic acid (9-AC, Sigma Aldrich, Germany). 9-AC is a ClC-1 blocker and its myotonia inducing effects have been confirmed *in vitro* and *in vivo* (Byrant and Morales-Aguilera, 1971; Tanimoto et al., 2012). A stock solution 9-AC was dissolved in dimethyl sulfoxide (DMSO). 9-AC stock solution was added to the solution to yield an end concentration of 100 μM.

### Muscle Dissection and Preparation

Wild type and BK^−/−^ mice aged between 45–125 days were killed by cervical dislocation after narcosis with CO_2_ for at least 2 min. The hindlimbs were dissected from the mouse trunk, and after moving the skin they were fixed in a large petri dish on a thin layer of Sylgard (Dow Corning, Belgium). The soleus (SOL) and extensor digitorum longus (EDL) muscles were dissected with the tendons intact from the hindlimbs of the animals in approximately 15 min under oxygenated Krebs-Ringer solution contained in the petri dish.

### Myographic Registrations

The muscle was mounted in an organ bath and was continuously bubbled with carbon (95% O_2_, 5% CO_2_, MTI IndustrieGASE, Neu-Ulm, Germany) as described previously (Hoppe et al., 2019). For muscle contractions experiments, physiological Krebs-Ringer solution was used containing the following (mM): 118 NaCl, 0.8 MgSO_4_, 1.0 KH_2_PO_4_, 11.1 glucose, 25 NaHCO_3_, and 2.5 CaCl_2_. One set of experiments was conducted using Bretag containing (mM): 107.7 NaCl, 0.69 MgSO_4_, 1.67 NaH_2_PO_4_, 5.05 Glucose, 26.2 NaHCO_3_, 1.53 CaCl_2_, 9.63 Na-gluconate, and 7.6 sucrose. KCl concentrations were adjusted to 4.5 mM and pH was set at 7.4. The temperature was maintained at 25°C for murine samples (Barclay et al., 2010). Each muscle specimen was attached to a highly sensitive force transducer (Model FT03, Glass Instruments, Quincy, MA, United States), coupled with a bridge amplifier and an analog-digital board (Digidata 1200B, Axon Instruments, Union City, CA, United States). A pair of platinum electrodes was placed on the lateral aspects of the muscle sample for electrical stimulation with supramaximal stimuli (25 V, 1 ms). To gain the optimum force development, muscle bundles were pre-stretched to approximately 150% of their initial length. All muscle samples were allowed to equilibrate in the chamber solution for at least 15 min prior to measurement.

**Figure 1:**
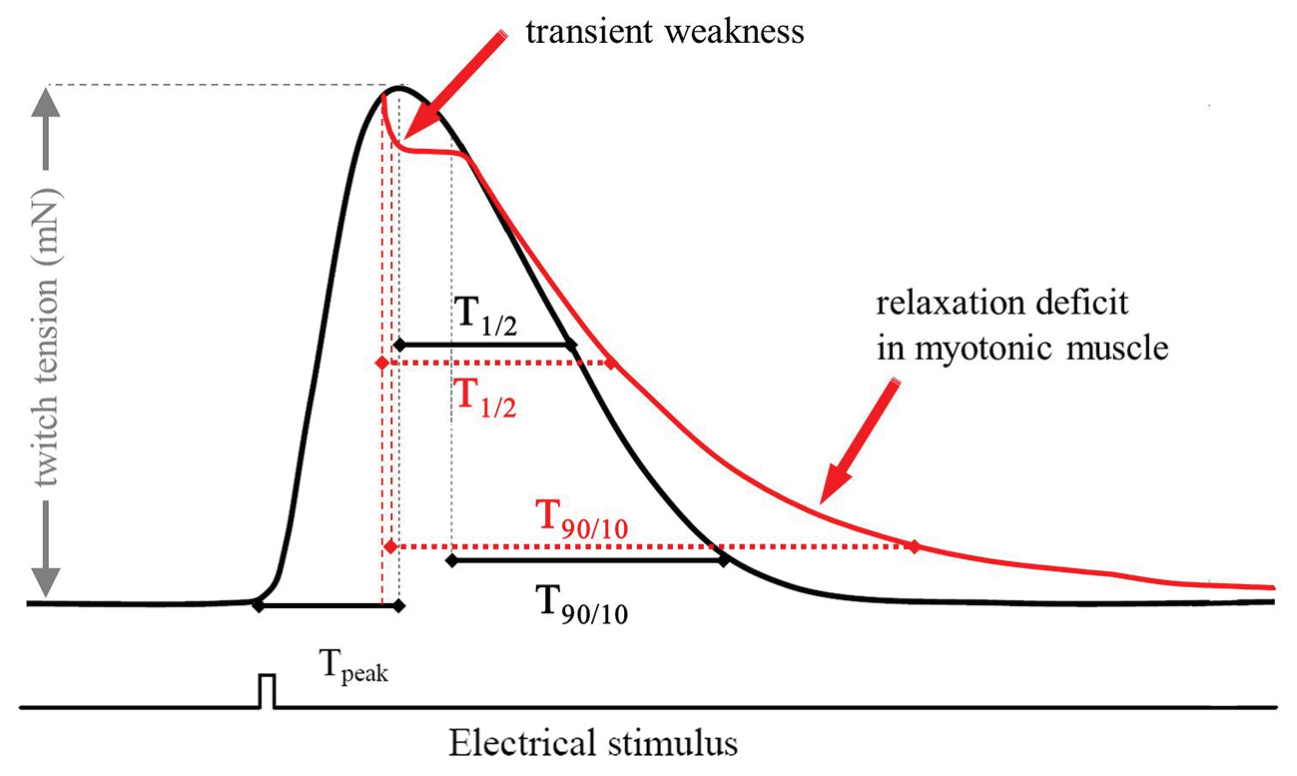
Scheme of a typical twitch recording with the corresponding parameters used for analysis. The red trace represents a typical recording of a myotonic muscle. The electrical stimulus has a duration of 1 ms and a voltage of 25 V. T_peak_ is a marker for the steepness of the upstroke of a single twitch. The relaxation times were measured as T_1/2_ (ms) – time to peak half and T_90/10_ (ms) – time from 90 to 10%.

For single experiments, muscle samples were stimulated at a frequency of 0.1 Hz. For each muscle a set of 20 successive twitches were recorded. The following contraction and relaxation parameters were evaluated: force (nM) – twitch tension, as the maximum force amplitude, T_peak_ (ms) – time to peak, measured from the beginning of the pulse until the twitch reached maximum amplitude, T_1/2_ (ms) – time to peak half. T_90/10_ (ms) – time from 90 to 10% of peak determined as the time between 90% of peak and the time when the force has decreased to its 10% value (ms; Figure 1).

Tetanic stimulation was characterized by following the protocol of the force-frequency relation. Different stimulation frequencies were applied for a fixed duration of 500 ms and the maximal force during each train was recorded. The maximal force during each frequency train was subsequently recorded and a set of two consecutive tetani were recorded for each muscle, with an interval of 60 s between recordings. For evaluation of transient weakness and the “warm-up” phenomenon, tetanic stimulation was prolonged to 4 s. The full “warm-up” frequency in myotonic muscle was determined as the frequency at which the relaxation deficit and transient weakness were absent.

### Pharmacological Substances Used in the Study

The BK channel activator NS1608 (Neurosearch, Ballerup, Denmark) and the blocker Paxilline (Sigma-Aldrich, Steinheim, Germany) were investigated on 9-AC induced myotonia of murine muscle. NS1608 as well as Paxilline were dissolved in 100% DMSO.

### Immunohistochemistry

Euthanized mice were perfused with 50 mM phosphate buffered saline (PBS; ice-cold), followed by 5% formalin in PBS for 10 min. EDL and SOL were removed, embedded in TissueTek OCT compound (Sakura) and quick-frozen over liquid nitrogen. Around 10 μm cryosections were thaw-mounted on poly-L-lysine-coated glass slides and stored at −20°C. For double immunofluorescence, samples were air-dried at room temperature, surrounded with RotiLiquid Barrier marker (Roth) and placed in a moist chamber. Samples were hydrated in 50 mM PBS and permeabilizided with 20% methanol in PBS followed by three changes (20 min each) of PBS/T (50 mM PBS with 0.2% Triton X-100). Protein blocking was conducted with 0.2% BSA and 10% NGS in PBS-T for 1 h. Samples were incubated with high affinity purified rabbit polyconal anti-BKα_(674–1,115)_ antibody (1:500) and mouse monoclonal anti-dihydropyridine receptor (DHPR) mAB_1A (Molecular probes) or anti-ryanodine receptor type 1 (RyR1)-mAB (Alexis Biochemicals), overnight at 4°C in PBS-T/0.2% BSA at 1 μg/ml each. Samples were washed three times with PBS-T (10 min each) and incubated with goat anti-rabbit Alexa 488 and goat anti mouse Alexa594 antibody (1:1000, Molecular Probes) for 90 min. Samples were washed with PBS. Barrier marker was removed mechanically, samples were covered with pPD-Glycerol (2 mg/ml, 80% glycerol in 50 mM PBS); coverslips were sealed with nail polish. Imaging was performed on a Zeiss Axioplon 2 imaging microscope equipped with a SPOT 7.0 monochrome camera (Diagnostic Instruments).

### Statistical Analysis

All data are presented as mean values (SEM). Potential differences between groups were evaluated using Wilcoxon matched-pairs signed-rank tests. Significance was set at a value of *p* < 0.05.

## Results

### Effects of Substances Influencing the BK Channel on Myotonic Murine Muscle Specimen

The BK channels are predominantly localized at the T-tubular membrane and are important for repolarization of the myofiber after an action potential. The BK channel activator NS1608 and the blocker Paxilline were investigated on 9-AC induced myotonia of murine muscle.

**Figure 2:**
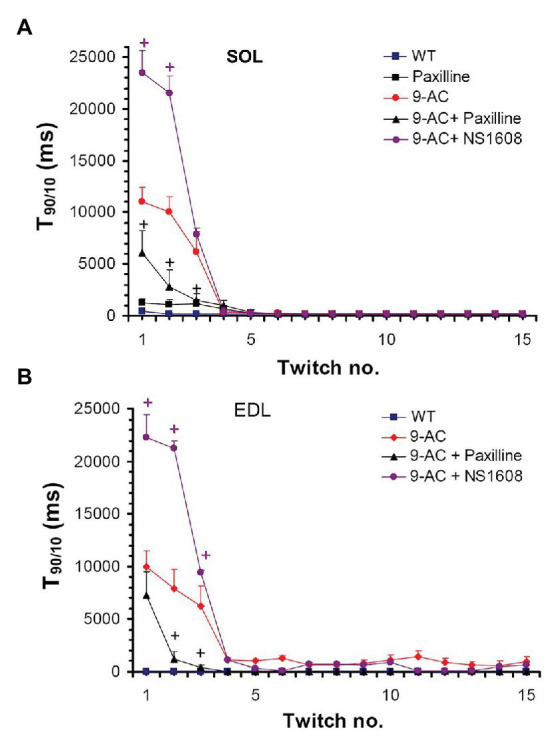
Effect of Paxilline and NS1608 on T_90/10_ in 9-AC induced low chloride conductance (gCl^−^) myotonia in murine muscle. T_90/10_ was recorded on six bundles of soleus (SOL; **A**) and extensor digitorum longus (EDL; **B**) isolated muscle after application of the BK channel blocker Paxilline (20 μM) or the BK channel activator NS1608 (20 μM). Paxilline caused a decrease in T_90/10_ in anthracene-9-carboxylic acid (9-AC; 100 μM) myotonic muscle whereas NS1608 had opposite effects. Paxilline in WT muscle caused a slight but not significant increase of relaxation time. Data displayed the T_90/10_ from the first 15 twitches in WT and 9-AC myotonic muscles. ^+^Significant difference vs. 9-AC.

Figure 2 demonstrates the effect on muscle relaxation time of 9-AC induced myotonia and BK channel modulators in WT Musculus SOL and EDL. The relaxation time (T_90/10_) kept stable over 15 consecutive contractions in WT muscle. In 9-AC treated muscle the relaxation deficit (T_90/10_) was more than 25 times prolonged compared to WT muscle. Application of Paxilline to wild-type SOL (WT-soleus, *n* = 4) caused a slight but not significant increase of T_90/10_ presumably through a delay in membrane repolarization (WT 380 ± 23; WT and Paxilline 1,190 ± 111) at twitch number one. In 9-AC treated WT-SOL and WT-EDL, 20 μM Paxilline significantly shortened the time of relaxation following contractions (SOL-9AC 11,080 ± 1,323; SOL-9AC and Paxilline 6,167 ± 2,289; EDL-9AC 10,080 ± 1,240; EDL-9AC and Paxilline 7,125 ± 2,623) at twitch number one. The application of 20 μM Paxilline to EDL-9AC muscle resulted in shortened relaxation times, which reached almost that of control (WT) muscle already after the second twitch. These results suggest that during muscle activity, an efflux of K^+^ through BK channels is involved in the development of myotonic stiffness.

Oppositely to Paxilline, 20 μM NS1608 effectively enhanced onset of myotonia as suggested by the aggravated myotonic activity of 9-AC leading to prolongation of T_90/10_ in the first muscle twitches (SOL-9AC 11,080 ± 1,323; SOL-9AC and NS1608 23,680 ± 2,210; EDL-9AC 10,080 ± 1,240; EDL-9 AC and NS1608 22,280 ± 2,381) at twitch number one.

### Effects of Substance Influencing the BK Channel on Myotonic Human Muscle Specimen

**Figure 3:**
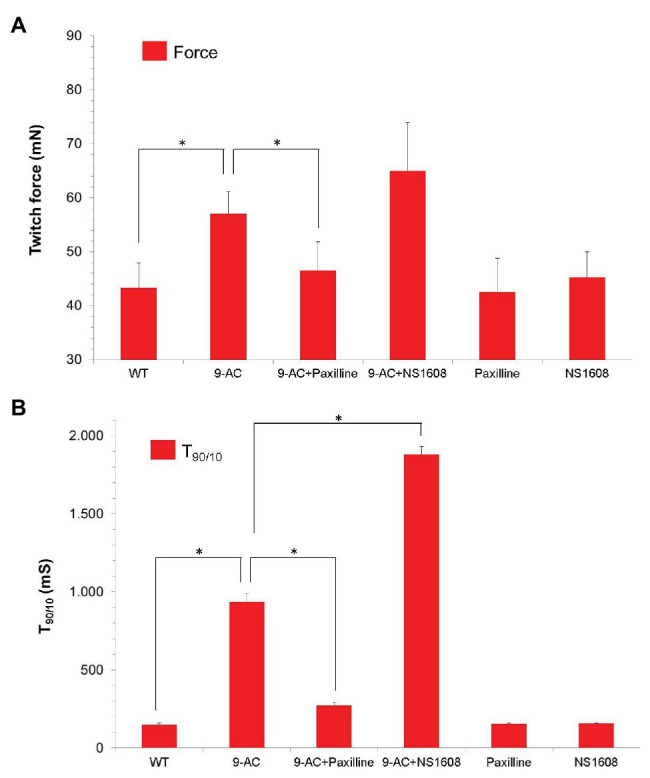
Effect of Paxilline and NS1608 in 9-AC induced low gCl^−^ myotonia in human samples. Data were obtained from the first twitch contraction in Musculus vastus lateralis obtained from healthy donors (*n* = 8). Low gCl^−^ myotonia was pharmacologically induced by 100 μM 9-AC in 4.5 mM [K^+^]_0_ KR. The BK^+^ channel blocker Paxilline (*n* = 4) or the BK^+^ channel opener NS 1608 (*n* = 4) were applied to 9-AC myotonic muscle at the concentration of 20 μM. Force **(A)** and T_90/10_ **(B)** were shown. Neither Paxilline nor NS1608 caused significant effects of relaxation times or force. Paxilline significantly reduced relaxation times T_90/10_ in 9-AC pretreated human muscle, while NS1608 significantly prolonged relaxation times in 9-AC pretreated human muscle. ^*^Significant difference vs. 9-AC.

To confirm the results of BK channel modifiers on murine muscle, NS1608 and Paxilline were also tested in human tissue from healthy donors (Musculus vastus lateralis; Figure 3). This muscle is characterized by a mixed fiber type composition (Kadi et al., 2006).

None of the compounds NS1608 and Paxilline significantly changed contraction and relaxation parameters in healthy human muscle specimens (T_90/10_: WT 149.2 ± 12.4; Paxilline 155.4 ± 7.5; NS1608 158.3 ± 3.1; Force: WT 43.3 ± 4.6; Paxilline 42.5 ± 6.3; NS1608 45.2 ± 4.8). In 9-AC treated human muscle the relaxation deficit (T_90/10_) was more than six times prolonged compared to WT muscle. In pharmacologically induced low chloride conductance (gCl^−^) myotonia, Paxilline and NS1608 significantly altered the myotonic activity in human muscle samples, as shown in Figure 3. Paxilline significantly reduced the relaxation time and the twitch force in human samples (T_90/10_: WT-9AC 936.0.2 ± 54.4; 9-AC-Paxilline 272.4 ± 20.43). Hence blocking the BK channel by Paxilline prevented the onset of myotonia. Again, NS1608 had the opposite effect to Paxilline by inhibiting muscle relaxation and aggravating myotonic activity (T_90/10_: WT-9AC 936.0.2 ± 54.4; 9-AC-NS1608 1881.3.4 ± 161.4). These findings in human muscle confirm those from the murine model of myotonia.

### Effects of Pharmacological Induced Myotonia by 9-AC in BK^−/−^ Mice

**Figure 4:**
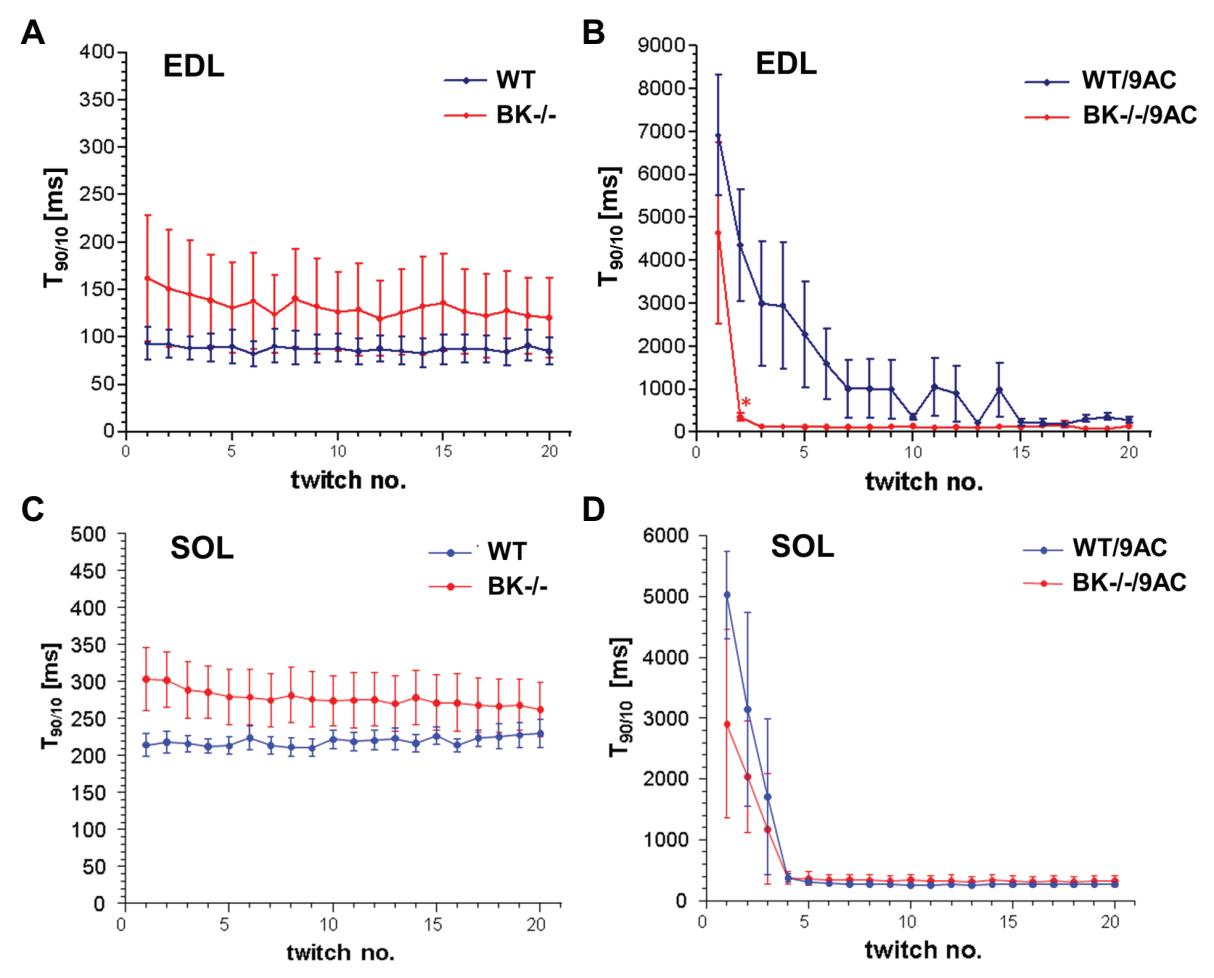
Effects of pharmacological induced myotonia by 9-AC in BK^−/−^ mice on T_90/10_. The effects of BK channels were evaluated in WT and BK^−/−^ muscle after induction of myotonia by 9-AC **(B)** or after application of control solution **(A)** on five bundles of EDL. Consecutively, WT and BK^−/−^ muscle were tested after induction of myotonia by 9-AC (*n* = 7; **D**) or after application of control solution (*n* = 5; **C**) on SOL muscle. Data displayed the T_90/10_ from the first 20 twitches in WT and BK^−/−^ after pharmacologically induced myotonia. ^*^Significantly different vs. WT.

**Figure 5:**
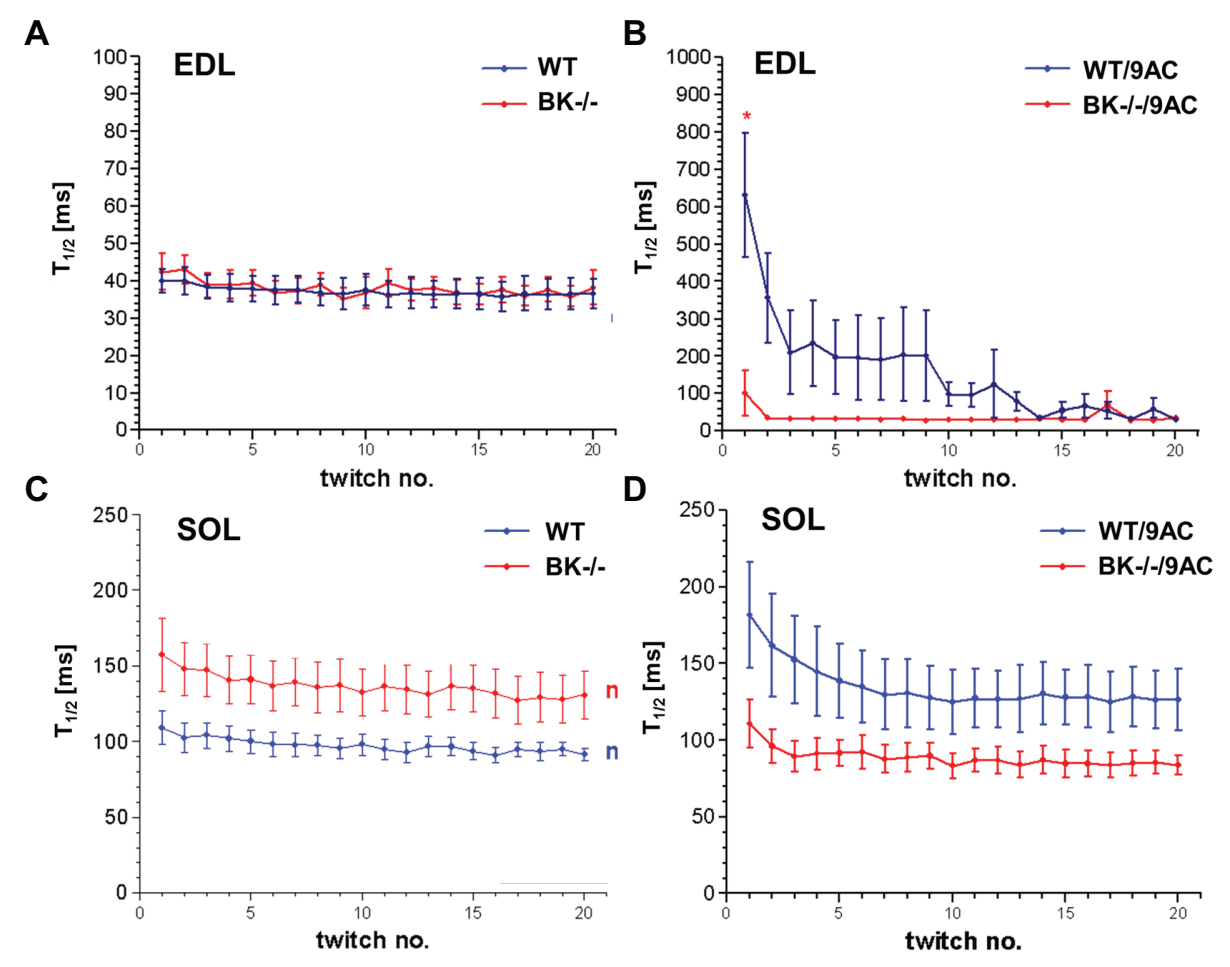
Effects of pharmacological induced myotonia by 9-AC in BK^−/−^ mice on T_1/2_. Half-time of relaxation (T_1/2_) for single twitches in EDL **(A,B)** and SOL **(C,D)** muscles from WT and BK^−/−^ mice in the presence of 9-AC **(B,D)**. Figures **(A,C)** represent T_1/2_ after application of control solution. Figures **(B,D)** represent T_1/2_ after induction of myotonia by 100 μM 9-AC. ^*^Significantly different vs. WT.

We next tested whether BK channel deficiency prevented the generation of myotonia by the ClC-1 blocker 9-AC. As demonstrated in Figures 4A,C relaxation times were slightly but not significantly prolonged in SOL and EDL muscle of BK^−/−^ mice compared to wild-type mice in pharmacologically untreated muscle (T_90/10_: EDL: WT 92.4 ± 19.2 BK^−/−^ 160.1 ± 68.2; SOL: WT 215.4 ± 18 BK^−/−^ 304 ± 40.9). Furthermore, we measured the muscle relaxation time of single twitches elicited by supramaximal stimuli in SOL and EDL muscle from BK^−/−^ mice and the respective littermates in myotonic muscle. While pronounced myotonia was generated by 9-AC in muscle of WT mice, relaxation times were shortened in muscle specimen of BK^−/−^ mice (T_90/10_: 1st twitch/2nd twitch: EDL: WT-9AC 6921 ± 1433/4396 ± 1,395; BK^−/−^-9AC 4689 ± 2034/320 ± 101; SOL: WT-9AC 5021 ± 780/3296 ± 1,598; BK^−/−^-9AC 2980 ± 1634/2092 ± 840; Figures 4B,D). In EDL muscle of BK^−/−^ mice the relaxation time of myotonic muscle induced by 9-AC reached that of non myotonic muscle already at the second twitch (Figures 4A,B). However, although the response was less pronounced in the SOL muscles, half-time of relaxation (T_1/2_) was decreased in BK^−/−^ soleus muscles in the presence of 9-AC (Figure 5D compared to 5C, conditions without 9-AC). In EDL muscle of BK^−/−^ mice, application of 9-AC resulted only in the first twitch to a slightly prolonged half-time of relaxation (T_1/2_; Figure 5B compared to 5A, conditions without 9-AC).

### Immunohistochemistry

**Figure 6:**
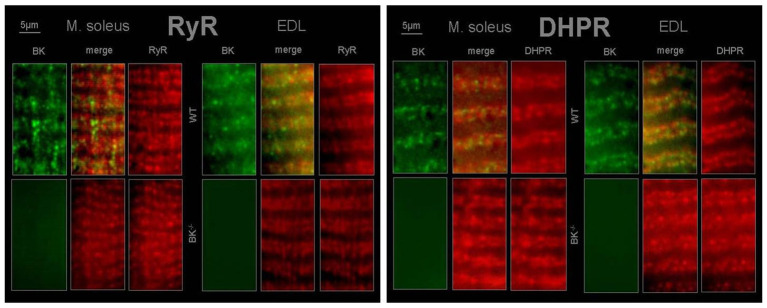
T-tubular co-localization of BK channels with dihydropyridine receptor (DHPR) and ryanodine receptor (RyR) SOL and EDL muscles were double immunofluorescence-labeled using a specific antibody against the BK channel (green) and the RyR type 1 (red) or DHPR (red). No staining for BK channels was observed in muscles from BK^−/−^ mice.

Immunohistochemistry revealed BK channels in close vicinity of RyR1 and the DHPR in EDL and less pronounced in SOL muscle of WT mice (Figure 6).

## Discussion

The main clinical symptom of MC is a deficit in relaxation (i.e., retained stiffness) after forceful voluntary contractions followed by transient weakness. Accumulation of K^+^ in the T-tubular system was suggested as a main contributing mechanism to cause the myotonic phenotype (Wallinga et al., 1999). Due to the low Cl^−^ permeability, the accumulation of [K^+^]_0_ in the T-tubular system is not compensated, finally resulting in a depolarization of the membrane (Adrian and Bryant, 1974; Clausen, 2013).

The BK channel is important for the repolarization phase after an action potential and dominantly localized on the T-tubular membrane. Local Ca^2+^ elevations *via* the Dihydrophyrdine receptor (DHPR) and the Ryanodine receptor channel (RYR) or large depolarization activate the BK_ca2+_ channel. Blocking of BK channels by Paxilline resulted in decreased relaxation times in murine and human muscle in pharmacologically induced myotonia, potentially due to an inhibition of K^+^ efflux, which is causative of T-tubular K^+^ accumulation and the electrical after-activity. These results were in agreement with increased relaxation times by the BK-agonist NS1608 in pharmacologically induced myotonia. Moreover, the data of this study show that muscle specimens of BK knock-out mice become less myotonic than those of WT mice when Cl^−^ channels are blocked in 9-AC solution. This is in agreement with the hypothesis that BK channel inhibition by Paxilline might result in a reduction of relaxation time in ADR (“arrested development of righting response” – an animal model for myotonia) muscle specimen as reported previously (Hoppe et al., 2020). However, apart from the function-degrading loss of CLC-1 channels, muscle fibers from human and animals with myotonia congenita have physiological, structural, and biochemical alterations, including hypertrophy (human, goats) or atrophy (mice) and alterations in fiber type composition (Beck et al., 1996; Jentsch et al., 2005). ADR mice are characterized by a generally impaired body condition with progressive muscle wasting, weakness, and force decline. To the best of our knowledge, this is the first investigation of BK channel modulators in pharmacologically induced myotonia in human and murine muscles.

BK channels were detected to localize to signaling complexes, which also contain calcium channels, or close to inositol 1,4,5-triphosphate (IP3) receptors and RYR (Grunnet and Kaufmann, 2004; Bentzen et al., 2014). As a consequence, it is difficult to predict the effect of BK channel activity in a given cell (Bentzen et al., 2014). However, BK channel activation will enhance efflux of potassium ions upon increased intracellular Ca^2+^. This causes a trend to hyperpolarization of the cell membrane resulting in decreased cell excitability (Bock and Stuart, 2016). Indeed, immunohistochemistry revealed BK channel localization in close vicinity to the voltage-sensing DHPR and the RYR in EDL and less pronounced in soleus muscle. Thus, from our results it can be hypothesized that (i) T-tubular activation of BK channels occurs by local Ca^2+^ elevations *via* DHPR and RYR and (ii) K^+^ is conducted by BK channels during the repolarizing phase of an AP and involved in the genesis of T-tubular K^+^ accumulation.

The BK-channel blocker Paxilline inhibited the development of myotonic stiffness in pharmacologically induced myotonia in human and murine muscle. The anti-myotonic effect of Paxilline may potentially be due to an inhibition of K^+^ efflux. Increasing [K^+^]_0_ triggered a dose-dependently depolarization of the resting membrane potential in ADR mice which caused a reduction of relaxation time (Hoppe et al., 2020). An inhibition of K^+^ efflux might, otherwise, reduce K^+^ accumulation in the T-tubular system and thus preventing subsequent after-potentials (Gu et al., 2007).

BK channels were originally reported to reduce the frequency of action potentials and to limit epileptiform bursts in neurons (Cannon et al., 1993; Gu et al., 2007; Bock and Stuart, 2016). However, BK channel blockade also slowed down the initial discharge frequency in response to current injection (Cannon et al., 1993; Gu et al., 2007; Bock and Stuart, 2016), an effect attributed to suppression of BK channel dependent rapid spike repolarization and fast after-hyperpolarization which both would be expected to increase inactivation of the spike-generating transient Na^+^ current and activate more of the slower K^+^ currents (Cannon et al., 1993; Gu et al., 2007; Bock and Stuart, 2016). Thus, BK channel blockade might result in enhanced refractoriness and reduced excitability (Latorre et al., 2000; Zhang et al., 2003). Additionally, Paxilline also inhibits the sarcoplasmic reticulum Ca^2+^ ATPases (SERCA) pump, which results in a decreased Ca^2+^ uptake into the sarcoplasmic reticulum and hence may influence muscle contraction (Bilmen et al., 2002). Apart from Ca^2+^ reuptake, SERCA pump inhibition was suggested to indirectly modulate Ca^2+^ release processes by determining the loading state of the internal Ca^2+^ stores (Santana et al., 1997). The activity of the RyRs as well as the inositol-1,4,5-triphosphate receptors (IP3Rs) are regulated by luminal sarcoplasmatic reticulum Ca^2+^ concentrations, which depend on SERCA pump activity (Missiaen et al., 1992; Sitsapesan and Williams, 1994). SERCA pump inhibition was suggested to decrease Ca^2+^ responses to agonists *via* RyRs and IP_3_Rs which resulted in decreased intracellular Ca^2+^ levels (Gòmez-Viquez et al., 2003) available for muscle contraction (Weisleder and Ma, 2008; Periasamy et al., 2017). The decreased intracellular Ca^2+^ levels may potentially reduce the open probability of BK channels resulting in an inhibition of K^+^ efflux, which is causative of T-tubular K^+^ accumulation and the electrical after-activity. However, exact mechanistic details remain to be clarified in future experiments, i.e., by applying a combination of calcium and action potentials measurements.

The BK channel activator NS1608 resulted in the prolongation of the relaxation deficit in pharmacologically induced myotonia. Moreover, BK channel activation entailed in a more rapid onset of the warm-up phenomenon, which may be at least in part mediated by the increased K^+^ level within the T-tubular system (Hoppe et al., 2020). These results support the role of BK channels in the regulation of T-tubular K^+^ accumulation.

Slow-twitch and fast-twitch skeletal muscle serve different motor functions, including postural maintenance and voluntary contractions. The phenotype of slow- and fast-twitch muscles can be distinguished by contractile proteins, cellular metabolism, or humoral regulation (Tricarico et al., 2005; Gao and Li, 2018). Moreover, these muscle types express BK channels that differ in their mean current amplitudes, current distributions, and Ca^2+^ sensitivity (Tricarico et al., 2005). While slow twitch myofibers contain mainly BK_slow_ with relatively low sensitivity to Ca^2+^ ions, fast-twitch myofibers are more sensitive to Ca^2+^ ions and relatively more responsive to carbonic anhydrase inhibitor (Tricarico et al., 2005). Experiments applying a rapid responding fluorescent Ca^2+^ indicator dye microinjected in murine muscle detected that the amount of calcium released is 3 to 4-fold larger in fast twitch fibers than in slow twitch fibers and the proportion of the released Ca^2+^ that binds to troponin to activate contraction is substantially smaller (Baylor and Hollingworth, 2012). The relatively low sensitivity of BK_slow_ to Ca^2+^ ions and the decreased level of free intracellular Ca^2+^ level in slow twitch muscle fibers compared to fast twitch muscle might at least in part explain the fact that myotonia is less prevented in soleus muscle of BK^−/−^ mice after pharmacological induction by 9-AC. Nevertheless, we were unable to detect differential effects of Paxilline or NS1608 after the pharmacological induction of myotonia in murine WT muscle specimens from either the predominantly slow-twitch soleus muscle or the primarily fast-twitch extensor digitorum longus muscle.

## Conclusion

The current “standard” therapy in myotonia congenita is ineffective in some individuals. Therefore the development of potential substances attenuating this muscle dysfunction is of note. This study points to a potential beneficial effect of the BK channel blocker Paxilline which caused an inhibition of myotonic stiffness. However, Paxilline is one of the most potent BK channel blockers and might be associated with side effects. It is a tremorgenic fungal alakaloid that causes tremors owing to its effects on BK channels of cerebellar purkinje cell (Chen et al., 2010). Further side effects (e.g. hyperactive bladder and increased cardiac injury after ischemia and reperfusion) would result from blockade of BK channels in other tissues (Sprossmann et al. 2009; Frankenreiter et. al. 2017). Based on the results of this study, further research investigating the effects of BK channel blockade is warranted in muscles of myotonic individuals.

## Data Availability Statement

The datasets generated for this study are available on request to the corresponding author.

## Ethics Statement

The studies involving human participants were reviewed and approved by Ethics Committee University of Ulm, Helmholtzstr. 16, 89081 Ulm. The patients/participants provided their written informed consent to participate in this study. The animal study was reviewed and approved by Animal Welfare Committee University of Ulm, Oberberghof, 89081 Ulm.

## Author Contributions

WK, FL-H, KJ-R, and KH were responsible for intellectual content and the study design. WK, TS, SC, FL-H, KH, and KJ-R were responsible for collection, analysis and interpretation of the data. KH, TS, WK, and SW were responsible for drafting the manuscript and graphical representation of the data. KJ-R, SW, TS, and WK were responsible for critical evaluation of the manuscript. All authors approved the final version of the manuscript submitted for publication. All persons designated as authors qualify for authorship and all those who qualify for authorship are listed.

### Conflict of Interest

The authors are not supported by, nor maintain any financial interests in, any commercial activities that may be associated with the topic of this article.
